# Efficacy and safety of human soluble thrombomodulin (ART-123) for treatment of patients in France with sepsis-associated coagulopathy: post hoc analysis of SCARLET

**DOI:** 10.1186/s13613-021-00842-4

**Published:** 2021-03-31

**Authors:** Bruno François, Maud Fiancette, Julie Helms, Emmanuelle Mercier, Jean-Baptiste Lascarrou, Toshihiko Kayanoki, Kosuke Tanaka, David Fineberg, Jean-Louis Vincent, Xavier Wittebole

**Affiliations:** 1 ICU Department, Inserm CIC-1435 & UMR-1092, CRICS-TRIGGERSEP, CHU Dupuytren, 2 Av Martin Luther King, 87042 Limoges, France; 2 Service de Médecine Intensive Réanimation, Centre Hospitalier Départemental Vendée, La Roche-sur-Yon, France; 3 Faculté de Médecine, Hôpitaux universitaires de Strasbourg, Service de Médecine Intensive - Réanimation, Nouvel Hôpital Civil, Université de Strasbourg (UNISTRA), Strasbourg, France; 4 Médecine Intensive Réanimation, CRICS-TRIGGERSEP, CHRU de Tours, Tours, France; 5 Service de Médecine Intensive Réanimation, Centre Hospitalier Universitaire de Nantes, Nantes, France; 6 Department of Program Management & Scientific Affairs, Asahi Kasei Pharma America Corporation, Waltham, MA USA; 7 Department of Medical Affairs, Asahi Kasei Pharma America Corporation, Waltham, MA USA; 8 Department of Intensive Care, Erasme Hospital, Université Libre de Bruxelles, Brussels, Belgium; 9 Department of Critical Care Medicine, Clinique Universitaire St Luc, UCLouvain, Brussels, Belgium

**Keywords:** Sepsis, Sepsis-associated coagulopathy, Thrombomodulin, ART-123, Heparin, France

## Abstract

**Background:**

The phase 3 multinational SCARLET study evaluated the efficacy and safety of a recombinant human soluble thrombomodulin (ART-123) for treatment of sepsis-associated coagulopathy (SAC), which correlates with increased mortality risk in patients with sepsis. Although no significant reduction in mortality was observed with ART-123 compared with placebo in the full analysis set (FAS), an efficacy signal of ART-123 was observed in subgroups of patients who sustained coagulopathy until the first treatment and those not administered concomitant heparin. Post hoc analysis was performed of patients treated in France, the country with the largest enrollment (19% of the FAS) and consistent patient enrollment throughout the study duration.

**Methods:**

Adult patients with SAC (international normalized ratio > 1.4; platelets > 30 × 10^9^/L to < 150 × 10^9^/L or platelet decrease > 30% within 24 h) and evidence of bacterial infection were included. The primary efficacy outcome was 28-day all-cause mortality. Safety outcomes included adverse, serious adverse, and major bleeding events. This analysis assessed patient characteristics and efficacy and safety outcomes in France compared with the rest of the world (ROW; excluding France). Mortality rates were assessed in patients in France or the ROW with characteristics previously associated with ART-123 efficacy.

**Results:**

Baseline characteristics were similar between France and the ROW, but some measurements of disease severity were higher in patients in France. The 28-day all-cause mortality absolute risk reductions (ARRs) with ART-123 were 8.3% in France and 1.1% in the ROW. The greater ARR in France may be related to a higher rate of sustained coagulopathy and lower rate of heparin use. In France and the ROW, 84.6% and 78.0% of patients sustained coagulopathy from the time of initial SAC diagnosis to first treatment with the study drug, and 65.8% and 43.9% did not receive heparin, respectively. The ARRs for these subgroups of patients in France were 13.4% and 16.6%, respectively. Safety of ART-123 was comparable between France and the ROW.

**Conclusions:**

Results from this exploratory analysis suggest that patients with sustained SAC not receiving concomitant heparin may benefit from ART-123, a fact that should be confirmed in future studies with more restrictive inclusion criteria.

**Supplementary Information:**

The online version contains supplementary material available at 10.1186/s13613-021-00842-4.

## Background

Sepsis is a life-threatening syndrome associated with multiorgan dysfunction that occurs when a host response to infection is dysregulated [1]. Sepsis-associated coagulopathy (SAC), characterized by an elevated international normalized ratio (INR) and reduced platelet count (PLT), correlates with an increased risk of mortality in patients with sepsis [2]. ART-123 is a recombinant human soluble thrombomodulin (thrombomodulin α) composed of 498 amino acids (64 kDa) from the soluble and active extracellular domains of thrombomodulin, and it exerts anticoagulation effects by binding thrombin and modifying its activity to amplify the formation of activated protein C [3, 4]. In addition, ART-123 exerts anti-inflammatory and antifibrinolytic effects through thrombin-activatable fibrinolysis inhibitor in the presence of thrombin [5].

Findings in a post hoc analysis of a phase 2b randomized clinical trial of participants with sepsis and suspected disseminated intravascular coagulation suggested that ART-123 (0.06 mg/kg per day for 6 days) may reduce mortality in patients with confirmed coagulopathy, indicated by prolongation of INR and reduction in PLT [6]. The SCARLET trial (NCT01598831) was a double-blind, randomized, multinational, placebo-controlled phase 3 study evaluating the efficacy and safety of ART-123 0.06 mg/kg per day for 6 days for treatment of SAC [4]. In the full analysis set (FAS), no statistically significant reduction in 28-day all-cause mortality—the primary efficacy endpoint of the study—was observed in patients who received ART-123 compared with patients assigned to placebo. The absolute risk reduction (ARR) of ART-123 versus placebo in the FAS was 2.55% [95% confidence interval (CI) − 3.68 to 8.77]. In subgroup analyses, ARRs with ART-123 versus placebo were 5.40% in patients who had sustained coagulopathy at baseline (from time of initial qualifying SAC diagnosis to time of first treatment with the study drug or placebo; *n* = 634; INR > 1.4 and platelet count > 30 × 10^9^/L) and 6.25% in patients not administered heparin (low-dose heparin for prophylaxis of deep venous thrombosis) before treatment with the study drug. Consistent with sustained coagulopathy, a subsequent post hoc analysis found larger ARRs in subgroups of patients with higher baseline levels of the coagulation biomarkers D-dimer, F1.2, and thrombin–antithrombin complex [7]. These analyses of SCARLET identified sustained coagulopathy at baseline and heparin administration as potential factors that could impact efficacy of ART-123 and could be modified to ensure the inclusion of patients most likely to benefit from ART-123 in future studies [8].

The SCARLET study was conducted in countries within Asia, Europe, North and South Americas, and the Asia-Pacific region [4]. These differences are hypothesized to have led to variability in study procedures, such as patient selection and concomitant treatments, and possibly patient outcomes among participating countries in the SCARLET study. Therefore, analysis of patients enrolled in one of the participating countries may be informative. Among 26 countries that enrolled patients in the study, France had the highest enrollment, accounting for 149 of 800 patients (19%) in the FAS. In addition, patient enrollment in France was consistent throughout the 5-year study period, a fact that may have limited confounding variables in study procedures related to timing and potentially contributed to consistency in patient outcomes. In this post hoc analysis, patient demographics, disease characteristics, treatment exposure, and efficacy and safety outcomes are reported in patients in France with SAC enrolled in the SCARLET study compared with the outcomes observed in participants from the rest of the world (ROW; excluding France).

## Methods

### Study design

Detailed methods of the SCARLET study have been previously published [4]. Briefly, patients aged ≥ 18 years with sepsis (defined by clinical objective evidence of bacterial infection and known site of infection, criteria for systemic inflammatory response syndrome of white blood cell count and temperature, and concurrent diagnosis of cardiovascular or respiratory dysfunction) and protocol-specified criteria for coagulopathy (INR > 1.4 without other known etiology and PLT > 30 × 10^9^/L to < 150 × 10^9^/L or PLT decrease > 30% within 24 h) were included. The INR values for each patient were generated using assays performed in laboratories of each center using mean prothrombin time values and corrected with the International Sensitivity Index. Eligible patients were randomized 1:1 to receive either intravenous ART-123 at a dose of 0.06 mg/kg/day (maximum dose of 6 mg/day) or equivalent placebo for ≤ 6 consecutive days. The primary efficacy endpoint of the SCARLET study was 28-day all-cause mortality after the first intervention. Primary safety endpoints included adverse events (AEs) and serious major bleeding events (SMBEs) through 28 days after the first dose of the study drug. A major bleeding event was defined as any intracranial hemorrhage, any life-threatening bleeding, any bleeding event classified as serious by the investigator, or any bleeding event that required administration of ≥ 1440 mL (typically 6 units) of packed red blood cells over 2 consecutive days.

This post hoc analysis explored efficacy and safety outcomes among the subgroups of patients enrolled in France (*n* = 149) and the ROW (*n* = 651). Baseline characteristics and additional factors reported during the SCARLET study were determined in each group. Efficacy was assessed with 28-day all-cause mortality, represented by the ARR of the ART-123 versus placebo groups. Patients in France and the ROW were further categorized on the basis of sustained coagulopathy (INR > 1.4 and platelets > 30 × 10^9^/L to < 150 × 10^9^/L) at baseline (between randomization and before administration of study drug) and administration of heparin after ICU admission.

Informed consent was received from all participants, their closest-blood relative, or legally authorized individual prior to performing any study-specific procedures to conform to all applicable local, regulatory, and ethical requirements. The SCARLET study was conducted in compliance with the International Conference on Harmonisation Good Clinical Practices [9]. The study was approved by appropriate ethics committees, as detailed in the previously published SCARLET study protocol [4].

### Statistical analyses

For comparisons between characteristics of patients in France versus the ROW, *P* values are based on either a *t* test or Chi-square tests depending on data type (continual or categoric, respectively). *P* values testing the equality of treatment effect between patients enrolled in France and the ROW are based on the interaction term of a logistic regression (i.e., the test for interaction of a region and treatment). *P* values < 0.05 were considered significant. Statistical analyses in this post hoc analysis were considered exploratory. *P* values did not represent known probability because they were neither prespecified nor controlled for multiplicity.

All calculations were performed using SAS version 9.4 (SAS Institute, Cary, NC, USA). Safety outcomes were summarized by descriptive statistics.

## Results

### Patient disposition and characteristics

**Fig. 1:**
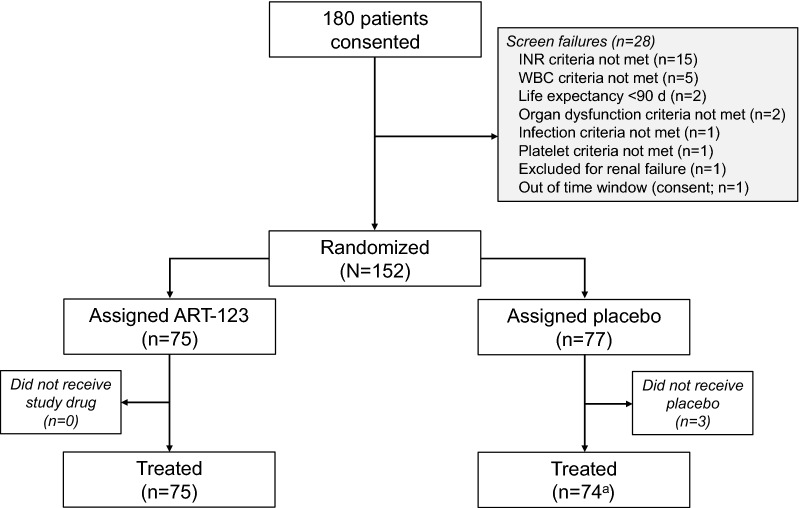
Patient disposition for the SCARLET Study in France. INR, international normalized ratio; WBC, white blood cell. ^a^One study patient randomized to the placebo group was administered a dose of ART-123 in error; this patient is included in the ART-1 23 group for safety analyses

There were 149 of 800 patients in the FAS enrolled in France from 17 sites, 75 of whom received ART-123 and 74 received placebo (Fig. 1). One patient randomized to the placebo group in France erroneously received a dose of ART-123 and is included in the ART-123 group for safety analyses. Of the 651 patients in the ROW, 320 patients received ART-123 and 331 patients received placebo (Additional file 1: Figure S1).

Among patients enrolled in France, patient demographics and characteristics were well balanced between the ART-123 and placebo groups (Table 1). Demographics of the total population of patients in France were generally similar to those of the patients in the ROW, including mean age and sex distribution. Nearly all patients enrolled in France and most patients in the ROW were White. Mean body mass index was ~ 27 kg/m^2^ in both France and the ROW, and the rates of diabetes were similar. Main causes for sepsis were abdominal and lung infections.

**Table 1:** Demographic and baseline characteristics of patients with SAC in France and ROW

Parameter	France			ROW
	ART-123 (*n* = 75)	Placebo (*n* = 74)	Total (*n* = 149)	Total (*n* = 651)
Age, mean (SD), year	63.7 (17.0)	63.7 (15.6)	63.7 (16.3)	60.0 (15.7)
Sex, *n* (%)				
Male	40 (53.3)	43 (58.1)	83 (55.7)	354 (54.4)
White ancestry, *n* (%)	73 (97.3)	72 (97.3)	145 (97.3)	475 (73.0)
BMI, kg/m^2^				
*n*^a^	–	–	–	649
Mean (SD)	27.6 (6.4)	26.3 (5.6)	27.0 (6.0)	26.7 (6.7)
Diabetes, *n* (%)	16 (21.3)	16 (21.6)	32 (21.5)	155 (23.8)
Total APACHE II score^b^				
*n*^a^	68	66	134	588
Mean (SD)	25.1 (7.4)	25.0 (6.9)	25.1 (7.1)	21.6 (8.1)
≥ 25, *n* (%)	36 (52.9)	35 (53.0)	71 (53.0)	212 (36.1)
Organ dysfunction^c^, *n* (%)				
*n*^a^			148	643
1	–	–	25 (16.9)	162 (25.2)
2			65 (43.9)	282 (43.9)
3 or 4			58 (39.2)	199 (30.9)
Arterial lactate				
*n*^a^	60	63	123	514
Mean (SD), mg/dL	42.7 (40.6)	36.0 (25.9)	39.3 (33.9)	35.0 (28.0)
> 55 mg/dL, *n* (%)	11 (18.3)	11 (17.5)	22 (17.9)	83 (16.2)
Creatinine				
*n*^a^	71	72	143	647
Mean (SD), µmol/L	166.9 (99.6)	183.5 (126.2)	175.3 (113.7)	154.2 (102.1)
Renal replacement therapy				
*n* (%)	3 (4.0)	6 (8.1)	9 (6.0)	84 (12.9)
Baseline heparin^d^, *n* (%)	21 (28.0)	30 (40.5)	51 (34.2)	365 (56.1)
Baseline coagulopathy^d,e^, *n* (%)	64 (85.3)	62 (83.8)	126 (84.6)	508 (78.0)

*APACHE II* Acute Physiology and Chronic Health Evaluation, *BMI* body mass index, *INR* international normalized ratio, *PLT* platelet count, *SD* standard deviation, *ROW* rest of world (excluding France)

^a^Total n for the parameter; data missing for some study patients

^b^Only applicable for study patients enrolled under protocol 2.0 or later

^c^Organ dysfunction was defined as: renal, creatinine > 2 µmol/L; liver, bilirubin ≥ 2 mg/dL; respiratory, on mechanical ventilation; cardiovascular, taking vasopressors

^d^After randomization and before treatment

^e^Coagulopathy was defined as INR > 1.4 without other known etiology and PLT 30 × 10^9^/L to 150 × 10^9^/L or PLT > 30% within 24 h

Mean Acute Physiology and Chronic Health Evaluation II (APACHE II) score of patients in France was significantly higher than the ROW (*P* < 0.0001; Table 1). Mean creatinine level was also significantly higher in patients in France (*P* = 0.04); however, the rate of renal replacement therapy was not significantly different. Arterial lactate concentrations and organ dysfunction (renal, hepatic, pulmonary, cardiovascular) were not significantly different between patients in France and the ROW.

The percentages of patients who had sustained coagulopathy (INR > 1.4 and platelets > 30 × 10^9^/L to < 150 × 10^9^/L) at baseline (from time of the initial qualifying diagnosis of SAC to time of first treatment with the study drug or placebo) were 84.6% and 78.0% in France and the ROW, respectively (Table 1). In addition, 65.8% of patients in France and 43.9% in the ROW did not receive heparin before treatment with the study drug.

### Treatment exposure

Patients in both France and the ROW had a mean treatment duration of approximately 5 days (Table 2). The majority of patients received 6 of the maximum 6 doses of the study drug, with no difference between France and the ROW. Time from ICU admission to administration of the first dose of the study drug was shorter for patients in France than it was for patients in the ROW (*P* < 0.0001). This included time between ICU admission and first qualifying diagnosis of SAC (INR > 1.4), a period that was also shorter in France compared with the ROW (*P* < 0.0001).

**Table 2:** Treatment exposure in France and ROW

Parameter	France (*n* = 149)	ROW (*n* = 651)	*P* value^a^
Duration of treatment, mean (SD), d	4.9 (1.86)	5.1 (1.68)	–
Dose, *n* (%)			
1	19 (12.8)	49 (7.5)	—
6	107 (71.8)	465 (71.4)	—
Time from admission to treatment			
*n*^b^	148	647	
Median (range), h	19.4 (12.3–26.8)	26.0 (17.9–41.3)	< 0.0001
Time from admission to qualifying INR			
*n*^b^	148	647	
Median (range), h	6.9 (0.7–17.0)	14.2 (4.5–29.6)	< 0.0001
Time from qualifying INR to treatment^c^, median (range), h	10.9 (6.9–14.8)	10.7 (7.0–14.5)	–

*INR* international normalized ratio, *ROW* rest of world (excluding France), *SD* standard deviation

^a^For comparison of patients in France vs those from the ROW by Wilcoxon 2-sample test

^b^Total n for the parameter; data missing for some study patients

^c^The study protocol limited the maximum time from qualifying INR to treatment

### Efficacy

The mortality ARRs were 8.3% (95% CI − 4.79 to 21.47) in France and 1.1% (95% CI − 5.87 to 8.16) in the ROW (Table 3); the difference in treatment effect did not reach statistical significance. The ARR for patients who had sustained baseline coagulopathy (*n* = 126) were 13.4% (95% CI − 0.97 to 27.79) in France and 3.2% (95% CI − 4.86 to 11.26) in the ROW (*n* = 508). An ARR of 16.6% (95% CI 0.40 to 32.77) was observed for patients in France who did not receive heparin before treatment with the study drug (*n* = 98), and an ARR of − 8.6% (95% CI − 32.62 to 15.47) was observed for those who received heparin (Table 3). In the ROW, patients who did not receive heparin (*n* = 286) had an ARR of 2.0% (95% CI − 8.65 to 12.74), and patients who did receive heparin in the ROW (*n* = 365) had an ARR of 0.4% (95% CI − 8.9 to 9.68). The treatment effect observed in patients in France who did not receive heparin was not significantly different from the same subgroup of patients in the ROW.

**Table 3:** Efficacy of ART-123 vs placebo in France and ROW

Mortality rate, %	Subpopulation	ART-123	Placebo	ARR (95% CI)
FAS	Global	26.8	29.4	2.55 (− 3.68, 8.77)
	France	17.3	25.7	8.3 (− 4.79, 21.47)
	ROW	29.1	30.2	1.1 (− 5.87, 8.16)
Baseline coagulopathy^a^	France	15.6	29.0	13.4 (− 0.97, 27.79)
	ROW	29.6	32.8	3.2 (− 4.86, 11.26)
No baseline heparin	France	13.0	29.5	16.6 (0.4, 32.77)
	ROW	29.7	31.8	2.0 (− 8.65, 12.74)

*ARR* absolute risk reduction, *CI* confidence interval, *FAS* full analysis set, *INR* international normalized ratio, *ROW* rest of world

^a^Coagulopathy was defined as INR > 1.4 without other known etiology and platelet count > 30 × 10^9^/L at baseline

### Safety

Rates of treatment-emergent AEs (any AE occurring after treatment with the study drug), treatment-emergent serious AEs, and on-treatment SMBEs were comparable among patients in France and the ROW and were similar between ART-123 and placebo groups (Table 4). The majority of patients in both France and the ROW experienced treatment-emergent AEs. Treatment-emergent serious AEs occurred in approximately one-half of patients in France and those from the ROW. Both populations had low rates of SMBEs.

**Table 4:** Safety of ART-123 in France and ROW

Event, n (%)	France			ROW
	ART-123 (*n* = 76^a^)	Placebo (*n* = 73)	Total (*n* = 149)	Total (*n* = 651)
TEAE	75 (98.7)	70 (95.9)	145 (97.3)	609 (93.5)
TESAE	38 (50.0)	36 (49.3)	74 (49.7)	334 (51.3)
On-treatment SMBE	5 (6.6)	4 (5.5)	9 (6.0)	30 (4.6)

*ROW* rest of world (excluding France), *SMBE* serious major bleeding event, *TEAE* treatment-emergent adverse event, *TESAE* treatment-emergent serious adverse event

^a^One patient randomized to the placebo group received a dose of ART-123

## Discussion

Although ART-123 did not significantly reduce mortality versus placebo in patients with SAC in France or the ROW, exploratory analyses of subgroups within the population of patients enrolled in France generally support the association of previously investigated factors with the efficacy of ART-123 [4]. The ARR with ART-123 versus placebo in the subgroup of patients from France who had sustained baseline coagulopathy (13.4%) exceeded the ARR observed in the total population of patients in France (8.3%) as well as the ARR of the same subgroup in the ROW (3.2%). In addition, ART-123 reduced mortality versus placebo in patients in France who did not receive heparin [ARR, 16.6% (95% CI 0.40 to 32.77)], and the ARR in this subgroup in France exceeded the ARR seen in the total population of patients in France (8.3%) as well as the ARR in the same subgroup in the ROW (2.0%). These trends are consistent with a previous analysis, which reported numeric increases in the ARRs of these subgroups among the FAS and provide rationale for further investigation [4]. For example, protocols for future testing of the efficacy of ART-123 could have stricter inclusion criteria for SAC diagnosis, only including patients with persisting coagulopathy, and exclude patients who receive heparin. Such studies could define specific subgroups for whom ART-123 might be an effective treatment, with the potential to reduce mortality rates in such patients with SAC.

Overall, 3 factors might have contributed to an efficacy signal of ART-123 in France compared with the ROW. First, a larger proportion of patients in France had sustained coagulopathy at the time of first treatment. Patients with sepsis who experienced normalization of coagulopathy before treatment may have been less likely than those with sustained coagulopathy to experience mortality related to SAC. Therefore, the population in France may have been enriched for patients with sustained coagulopathy and, thus, a greater likelihood to benefit from ART-123 treatment. Second, a smaller proportion of patients in France compared with the ROW received concomitant heparin. Third, patients in France experienced a significantly shorter time from ICU admission to first qualifying SAC diagnosis versus the ROW, thereby resulting in an overall shorter time to treatment. This observation may have contributed to the higher rate of patients who had sustained coagulopathy at the time of the first treatment and also may have directly impacted the efficacy signal of ART-123, because patients received treatment sooner.

The organization of ICUs varies among countries and may impact patient outcomes. Most ICUs in western Europe are “closed” and have a dedicated intensivist on call to care for admitted patients. The closed ICU model may be associated with lower mortality rates and a shorter length of stay [10, 11]. Intensivists in France are also actively involved in clinical research, as evidenced by the high participation of French sites in the SCARLET study and existence of a sepsis clinical research support network in France.

One could speculate that having a dedicated intensivist and a team experienced in clinical research could inform study procedures such as patient monitoring and treatment times. Although not all disease markers were significantly different between the populations, patients from France had higher APACHE II scores and creatinine levels than those seen in the ROW, potentially reflecting differences in patient selection. Patients in France also experienced shorter median times from ICU admission to SAC diagnosis, resulting in an overall shorter median time to treatment. The timely selection, diagnosis, and treatment of patients critically ill with SAC may have contributed to the larger percentage of patients who still presented with protocol-specified coagulopathy at the time of their treatment and lower rate of heparin use, supporting a research environment in France being more sensitive to detect a signal for ART-123 efficacy.

Notably, some patients in the SCARLET study experienced prompt normalization of SAC. We speculate that this observation may have been associated with response to early standard of care interventions. For these patients, their condition improved during the screening period and their coagulation parameters normalized before the blood draw at the time of the first study treatment (baseline).

A limitation of this post hoc analysis is that it was underpowered to determine statistical significance in the comparisons between the patients enrolled in France and those enrolled in the ROW and within exploratory subgroups. The authors also acknowledge the limitations of INR expression of prothrombin time in patients not receiving vitamin K agonist treatment. However, data from the analysis provide rationale for the pursuit of additional studies sufficiently powered to identify patient populations with SAC for whom ART-123 may provide clinical benefit.

## Conclusions

It was hypothesized that variability among the patient populations and ICUs in the participating countries could potentially be associated with efficacy and safety outcomes during the SCARLET study. Therefore, post hoc analysis was performed to determine the efficacy of ART-123 in France, the country with the highest study enrollment and whose enrollment remained steady through the 5-year study period. The present study describes a similar safety profile of ART-123 in patients from France and the ROW. Post hoc analysis of 28-day all-cause mortality rates identified a numerically larger ARR for patients who participated in the study in France (8.3%) compared with those from the ROW (1.1%) and explored certain factors potentially associated with the efficacy of ART-123 in this patient population. In particular, the authors believe that the efficacy of ART-123 may be higher in patients with an earlier and persistent SAC diagnosis and in those not receiving concomitant heparin. Thus, results from this exploratory analysis provide data that may help to better identify patients with SAC likely to benefit from ART-123.

## Supplementary Information

**Additional file 1: Figure S1.** Participating countries and patient and disposition for the SCARLET Study in the Rest of the World (excluding France).

## Data Availability

The dataset analyzed in the current study is unavailable because the informed consent form signed by patients did not address an individual data-sharing statement. General Data Protection Regulation prohibits disclosure of individual data in the European Union. In this situation, we are unable to share the individual data of this trial.

## Abbreviations

AE: Adverse event
APACHE II: Acute Physiology and Chronic Health Evaluation
ARR: Absolute risk reduction
BMI: Body mass index
CI: Confidence interval
FAS: Full analysis set
ICU: Intensive care unit
INR: International normalized ratio
PLT: Platelet count
ROW: Rest of world (excluding France)
SAC: Sepsis-associated coagulopathy
SD: Standard deviation
SMBE: Serious major bleeding event
TEAE: Treatment-emergent adverse event
TESAE: Treatment-emergent serious adverse event
WBC: White blood cell
